# Hypertrophic Cardiomyopathy Mimicking Acute Anterior Myocardial Infarction Associated with Sudden Cardiac Death

**DOI:** 10.1155/2012/236154

**Published:** 2012-08-16

**Authors:** Y. Daralammouri, M. El Garhy, K. Same, B. Lauer

**Affiliations:** Department of Cardiology, Heart Center, Zentralklinik Bad Berka, Robert-Koch-Allee 9, 99437 Bad Berka, Germany

## Abstract

Hypertrophic cardiomyopathy is the most common genetic disease of the heart. We report a rare case of hypertrophic obstructive cardiomyopathy mimicking an acute anterior myocardial infarction associated with sudden cardiac death. The patient presented with acute ST elevation myocardial infarction and significant elevation of cardiac enzymes. Cardiac catheterization showed some atherosclerotic coronary artery disease, without significant stenosis. Echocardiography showed left ventricular hypertrophy with a left ventricular outflow tract obstruction; the pressure gradient at rest was 20 mmHg and became severe with the Valsalva maneuver (100 mmHg). There was no family history of sudden cardiac death. Six days later, the patient suffered a syncope on his way to magnetic resonance imaging. He was successfully resuscitated by ventricular fibrillation.

## 1. Introduction

**Figure 1:**
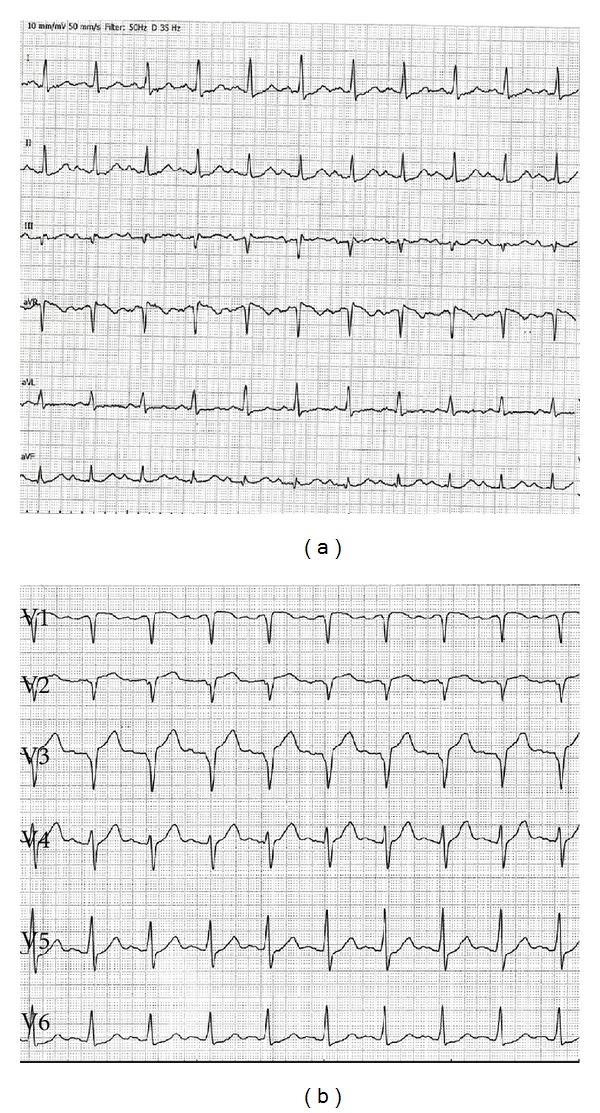
Standard 12-lead ECG shows sinus rhythm at 120 bpm, poor R wave progression in V1–V3 leads, an increased S wave in V1–V3 associated with ST segment elevation in V1–V3.

**Figure 2:**
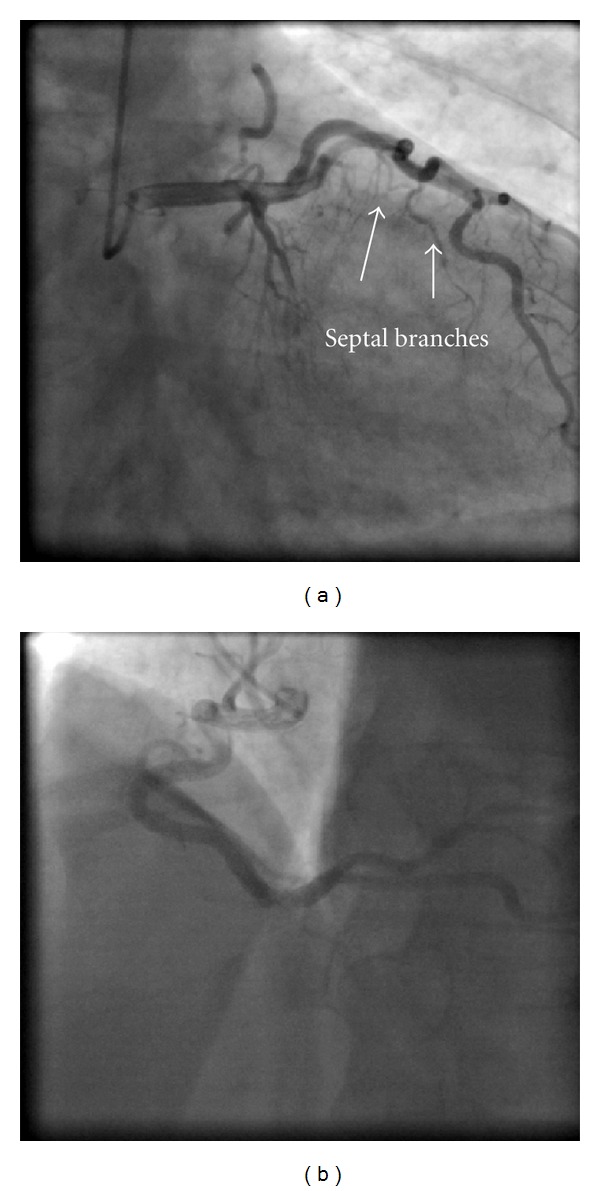
A coronary angiogram shows coronary fistula from the left anterior descendens (LADs) into the pulmonary artery (a) without significant coronary stenosis. (b) Right coronary artery.

**Figure 3:**
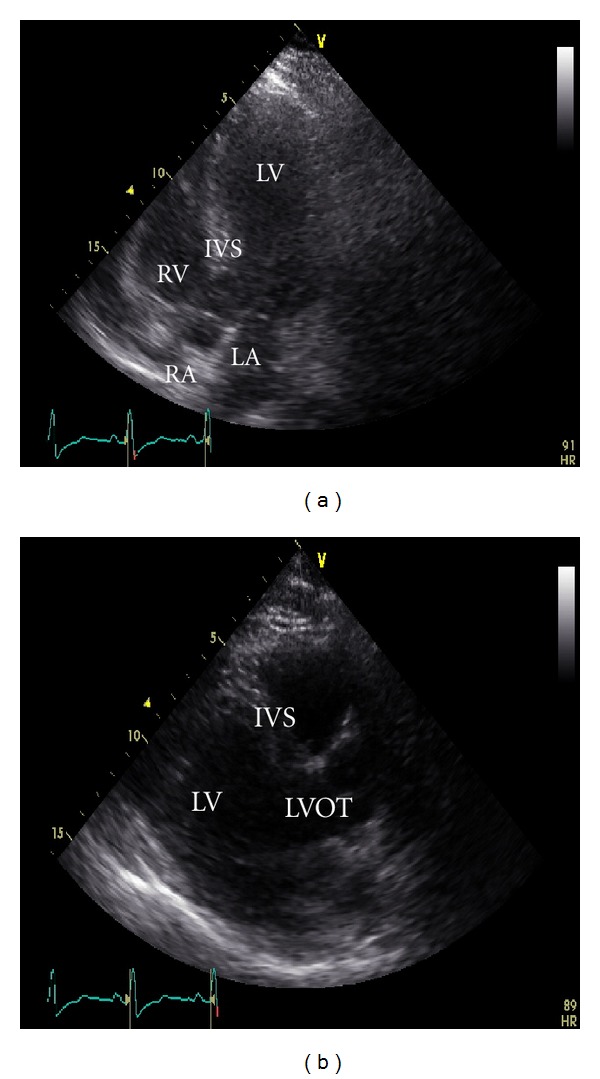
Apical four-chamber view (a) and parasternal long axis view (b) with poor acoustic window revealed thickened left ventricular walls. LV: left ventricle; LA: left atrium; LVOT: left ventricular outflow tract; RV: right ventricle; RA: right atrium; IVS: interventricular septum.

**Figure 4:**
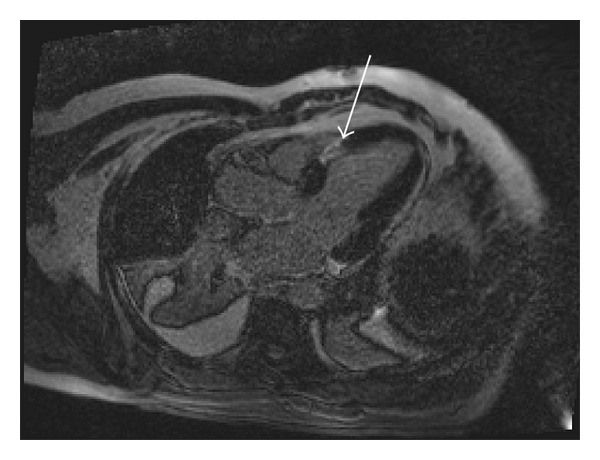
CMR (long axis) shows delayed enhancement in the interventricular septum (arrow).

A 45-year-old male patient presented to our emergency room complaining of recurrent chest pain at rest (CCS IV). Past medical history was noncontributory except for hypertension and smoking. The patient was not on any regular medication. The patient's vital signs included the following: blood pressure was 160/100 mm Hg, resting heart rate was 120 beats/min, respiratory rate was 18 breaths/min, oxygen saturation was 98%, and temperature was 37.0°C. Cardiac auscultation revealed normal first and second heart sounds and 2/6 systolic murmur over the apex. There were no congested neck veins. Neither lower limb edema nor signs of pulmonary congestion were observed. The initial ECG showed ST elevation in the precordial leads from V1 to V3 and poor R wave progression in V1–V3 leads V1-3 (Figure 1). The initial diagnosis of acute coronary syndrome (ST elevation myocardial infarction) was established, and the patient was immediately transferred to our catheter lab. A coronary angiogram, however, showed some atherosclerotic coronary artery disease without significant stenosis (Figures 2(a) and 2(b)). Left ventriculography demonstrated a normal ejection fraction (estimated to be approximately 65%) without regional wall motion abnormalities. Pressure tracings showed a pressure gradient between the *left ventricular* myocardium LV and the aorta with a peak to peak of 20 mm Hg. The patient's complete blood count, basic metabolic panel, and liver function tests were all within the normal range. Two sets of myocardial enzyme assays showed a progressive increase in creatine phosphokinase levels from 17.5 *μ*mol/s/L to 19 *μ*mol/s/L (normal range, <5.14 *μ*mol/s/L) and troponin T levels from 1.0 ng/mL to 1.5 ng/mL (normal range, 0–0.01 ng/mL) during the first 6 hours after admission. After 3 days, echocardiography revealed normal LV systolic function, no resting wall motion abnormalities, LV septal wall thickness of 21 mm (Figure 3), and systolic anterior motion of the anterior mitral valve leaflet. There was a peak systolic pressure gradient between the LV and ascending aorta of 20 mm Hg at rest, increasing to 100 mmHg after the Valsalva maneuver. Telemetric monitoring showed no abnormalities for 72 hours, and therefore, the patient was transferred to the ward. On day 6, the patient was found unconscious on his way to the cardiac- magnetic resonance imaging (CMR). He was successfully reanimated, with defibrillation by ventricular fibrillation. CMR showed severe late gadolinium enhancement in the interventricular septum (Figure 4). We implanted an implantable cardioverter-defibrillator (ICD) for secondary prophylaxis by hypertrophic obstructive cardiomyopathy (HOCM). The patient was discharged on medical treatment including a *β*-blocker. Upon followup after 3 months, the patient was asymptomatic, whereas transthoracic echocardiography continued to show the same gradient of 20 mmHg across the left ventricular outflow tract obstruction (LVOT). 

## 2. Discussion

Hypertrophic cardiomyopathy (HCM) is the most common genetic disease associated with more than 1000 mutations in 11 genes [1]. HCM is caused by an autosomal dominant mutation in genes that encode sarcomere proteins or sarcomere-associated proteins [2]. Evidence shows that 8 genes are known to definitively cause HCM: beta-myosin heavy chain, myosin-binding protein C, troponin T, troponin I, alpha tropomyosin, actin, regulatory light chain, and essential light chain [3–7]. Most patients have obstruction of the left ventricular outflow tract (LVOT) at rest or after physiological provocation [8]. HCM is associated with cardiac sudden death (SCD). The mortality rate is approximately 1% [9]. HCM is the most common cause of SCD in young people [10]. In 75% to 95% of HCM patients, ECG shows changes in the form of left ventricular hypertrophy [11, 12]. The clinical diagnosis of HCM is conventionally made with cardiac imaging, most commonly with 2-dimensional echocardiography and increasingly with CMR [2]. A negative inotropic medication, such as a *β*-blocker or nondihydropyridine calcium channel blocker, is the most appropriate initial therapeutic intervention. Both *β*-blockers and calcium channel blockers can decrease the obstructive gradient in HCM by decreasing catecholamine-mediated contractility [13]. Dual-chamber pacing has been used in drug-refractory HCM. Right-ventricular pacing with a short atrioventricular delay results in dyssynchronous LV contraction and reduces LVOT obstruction [14]. An ICD is the mainstay therapy for SCD prevention and is a class I indication for secondary prevention in patients with a history of ventricular fibrillation or hemodynamically unstable ventricular tachycardia [2]. Contrast-enhanced CMR with assessment of late gadolinium enhancement (LGE) to identify areas of myocardial fibrosis or scarring in patients with HCM has been used to risk stratification for SCD [15, 16]. Acute dynamic LVOT obstruction elevates left ventricular filling pressure, increasing myocardial oxygen demands, and ultimately leads to ischemia. In some patients, this situation can cause a transient left ventricular apical ballooning [17, 18]. In our case, we did not observe this situation. We hypothesize that acute dynamic LOVT obstruction in our case was the initial mechanism of acute chest pain. In addition, myocardial ischemia and the use of nitrates, in our emergency room, which worsened the LVOT gradient, caused further clinical deterioration. There are another mechanisms could explain this myocardial necrosis such as thromboembolic event to the septal branch or coronary spasm. Coronary spasm sometimes accompanies with hypertrophic cardiomyopathy. CMR imaging demonstrated a severe LGE in the interventricular septum and this suggested that the myocardium was prone to ventricular tachyarrhythmia.
